# Health Benefits of Supplementing Nursery Pig Diets with Microalgae or Fish Oil

**DOI:** 10.3390/ani9030080

**Published:** 2019-03-05

**Authors:** Alison V. Lee, Lan You, Se-Young Oh, Ziwei Li, Alexandra Code, Cuilan Zhu, Rebecca E. Fisher-Heffernan, Timothy R. H. Regnault, Cornelis F. M. De Lange, Lee-Anne Huber, Niel A. Karrow

**Affiliations:** 1 Department of Animal Biosciences, University of Guelph, ON N1G 2W1, Canada; alee09@uoguelph.ca (A.V.L.); youlan0803@gmail.com (L.Y.); ugohs530@gmail.com (S.-Y.O.); zli05@uoguelph.ca (Z.L.); acode@uoguelph.ca (A.C.); czhu@uoguelph.ca (C.Z.); rfisher01@gmail.com (R.E.F.-H.); cdelange@uoguelph.ca (C.F.M.D.L.); huberl@uoguelph.ca (L.-A.H.); 2 Department of Obstetrics and Gynecology and Physiology and Pharmacology, Western University, London, ON N6A 5C1, Canada; tim.regnault@uwo.ca

**Keywords:** microalgae, fish oil, swine nutrition, lipopolysaccharide, acute-phase response, immune response

## Abstract

**Simple Summary:**

Weaning is a stressful event and the associated stress can affect piglet’s growth and health. The inclusion of omega-3 polyunsaturated fatty acids (n-3 PUFA) in piglets’ diets may reduce the inflammation associated with stress occurring at weaning, allowing for optimal growth and health. Many n-3 PUFA sources are fish-based; however, the use of microalgae may provide a similar alternative to fish products. We therefore investigated the use of fish oil or microalgae in piglets’ diets in addition to less expensive plant-based protein sources and assessed the effects of piglets’ diet on growth and health. It was determined that the inclusion of fish oil or microalgae did not affect piglet’s growth, but dietary fish oil reduced feed intake when pigs were placed on a common diet. Microalgae and fish oil supplementation also decreased the stress response following an immune stress challenge. However, no effects of piglet’s diet were found on piglet’s immune response. Results from this trial suggest that microalgae and fish oil can differentially affect the piglet’s stress response, possibly due to different nutrient profiles in the two feed ingredients.

**Abstract:**

Weaning stress can negatively impact a pig’s performance; dietary supplementation with omega-3 polyunsaturated fatty acids (n-3 PUFA) reduces inflammatory stress and promotes nursery pig’s health and growth. Fish oil (FO) is a major source of n-3 PUFA; however, microalgae (AL) may provide an alternative source of n-3 PUFA. The aim of this study was to assess the health benefits of supplementing a plant protein-based nursery diet with 3.12% AL or 1.25% FO providing equal total n-3 PUFA compared to a control (CON) diet. Seventy-two pigs were fed experimental diets for three weeks (phases 1 and 2), followed by a common standard diet for three weeks (phase 3). Following phase 2, 8 pigs per treatment underwent a lipopolysaccharide (LPS) immune stress challenge to assess the acute-phase response and 8 pigs per treatment were vaccinated with novel antigens to assess acquired immunity. No significant differences in piglets’ growth were observed, despite decreased feed intake in FO piglets compared to AL piglets in phase 3. AL supplementation tended to reduce, and FO supplementation significantly reduced the LPS-induced fever response. The AL pigs had significantly reduced cortisol responses, increased cytokine concentrations, and increased chromogranin A concentrations compared to FO and CON pigs following LPS challenge. Results suggest that AL or FO supplementation in nursery diets differentially modulate the acute-phase response, possibly due to different n-3 PUFA profiles between the two ingredients.

## 1. Introduction

Peri-weaning mortality and morbidity are significant concerns and important welfare issues for the swine industry. The stress of weaning results in reduced feed intake the week following weaning, and this can negatively impact growth [1]. In addition, the stress of weaning and subsequent co-mingling of animals can leave pigs vulnerable to secondary stressors, such as microbial infection, that can influence life-time productivity [1]. Historically, in-feed antibiotics were used to combat post-weaning growth check and post-weaning disease challenges; however, this is now discouraged due to concerns of antimicrobial resistance [2]. Therefore, alternative feeding strategies are being investigated to promote pig growth and health. For example, dietary supplementation with ingredients rich in omega-3 polyunsaturated fatty acids (n-3 PUFA) may reduce gut and systemic inflammation that can occur during the transition from sow milk to solid feed. When this strategy was explored using fish oil (FO) in weaner pigs’ diets, inclusion of FO improved growth and immunity compared to diets with the same caloric value having lower n-3 PUFA content [3].

The stress of weaning also includes adjusting to solid feed. Post-weaning diets containing high-quality protein sources are thought to promote growth and health, despite weaning stress-associated reductions in feed intake. However, high-quality protein diets are expensive, due to the inclusion of highly digestible products such as whey protein. Soybean meal (SBM) is a commonly included feed ingredient in swine diets; typically containing 48% crude protein (CP), SBM is a comparatively less expensive protein source, in part due to its amino acid composition and digestibility [4]. A previous study found that feeding diets with a typical low-quality SBM protein source during the nursery phase had no negative consequence on the final market weight of pigs [5]. However, it has also been found that when faced with sub-clinical infection, piglets on a low-quality protein diet have reduced growth performance compared to uninfected piglets on the same diet [6]. SBM is also known to have allergenic components that can contribute to gut inflammation [7]; this can negatively impact piglet growth and could increase their susceptibility to disease [1].

Due to increased demand for FO and decreased environmental sustainability of fish products for both human and animal health, there is a need to find alternatives to FO that have similar health benefits and functions: one plausible alternative is microalgae (AL) containing n-3 PUFA. Using AL in nursery piglets’ diets containing simple SBM protein sources could facilitate the use of less expensive protein sources in nursery diets without compromising piglets’ growth or health status.

Therefore, the aim of this study was to examine the effect of AL and FO inclusion in a post-weaning pig’s diet containing a typical, low-quality SBM protein source, and to measure growth performance and neuroendocrine-immune biomarkers as a measure of health following either immune stress challenge with lipopolysaccharide (LPS), or vaccination with novel antigens. It was hypothesized that when FO and AL diets were matched for the total n-3 PUFA content, no differences would be observed between these treatments, supporting the use of AL as an alternative to FO.

## 2. Materials and Methods

The experimental protocol (Animal Utilization Protocol # 3124) was approved by the University of Guelph Animal Care Committee and followed Canadian Council of Animal Care guidelines (CCAC, 2009). The study was conducted at the Arkell Swine Research Station at the University of Guelph (Guelph, ON, Canada). The vitamin E and the Menhaden FO for this experiment were provided by Grand Valley Fortifiers (Cambridge, ON) and the AL (All-G-Rich, *Aurantiochytrium limacinum* dry biomass (AURA; CCAP 4087/2) containing 15.8% crude protein, 70% crude fat and 17% docosahexanoic acid) was provided by Alltech Inc. (Nicholasville, KY). Fatty acid analysis was conducted on the FO and AL at the University of Guelph Lipid Analytical Laboratories by the method as described below. Phase 1 and 2 nursery diets were analyzed for dry matter, crude protein, crude fat, and macromineral content by SGS laboratories Canada (Guelph, ON).

### 2.1. Piglet Experimental Procedure

Seventy-two piglets (Landrace x Yorkshire, 36 females and 36 castrated males) were selected, weaned at 21 days of age, and randomly allocated to one of three dietary treatments: a low-quality protein corn- and SBM-based diet supplemented with either 1.25% FO or 3.12% AL, and 5% corn oil as a control (CON). Inclusion of FO was selected based on a previous research conducted by Huber et al. [3], and inclusion level of AL was selected to match the total n-3 PUFA content of the FO diet. Percentage of crude fat (CF), docosahexanoic acid (DHA), eicosapentanoic acid (EPA), and total omega-3 content of the AL and FO ingredients are included in Table 1. Piglets were grouped into pens with 6 pigs per pen (3 females and 3 castrated males) and 4 pens per dietary treatment. Initial body weight was 6.86 ± 0.25 kg for all pigs, and did not differ across pens or treatments. The diets were formulated to meet the recommended nutrient requirements [4] for nursery phases 1 (d0–7 post-weaning), 2 (d7–21), and 3 (d21–42; Table 2). The phases 1 and 2 FO and AL diets were matched for total n-3 PUFA content. After d21, all piglets were placed on a common phase 3 control diet for the remainder of the trial (d21–42). After nursery phase 1, 2 pigs (1 female and 1 castrated male) were randomly selected and were removed from each pen and transported to the University of Guelph for LPS challenge (see below), leaving a total of 4 pigs per pen. Feed and water were provided *ad libitum*. The pigs were weighed weekly to calculate the average daily gain (ADG) per pen, and feed disappearance was recorded and divided by the number of piglets per pen to calculate the average daily feed intake (ADFI) and gain-to-feed ratio (G:F). All pigs were used for performance analysis in phase 1; only pigs that remained at the Arkell swine research station were included in the performance analyses for phases 2, 3, and overall.

### 2.2. Assessment of Acute-Phase Response to LPS Immune Stress Challenge

**Figure 1:**
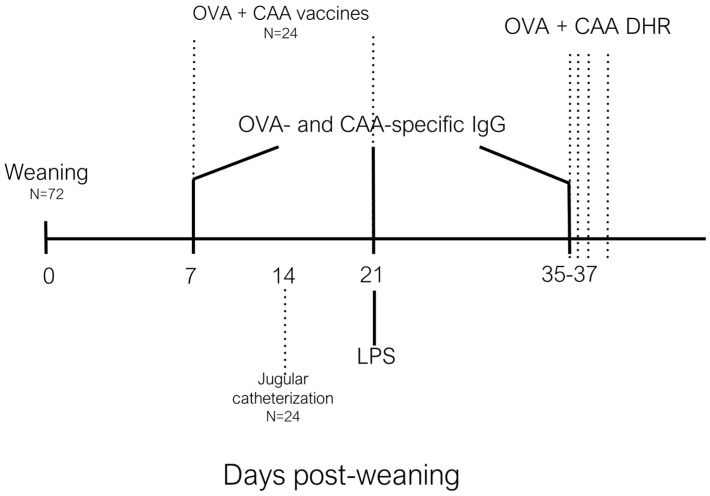
Schematic timeline of trial events. OVA, ovalbumin; CAA, *Candia albicans* antigen; LPS, lipopolysaccharide; IgG, immunoglobulin G; DHR, dermal hypersensitivity response.

On day 7 post-weaning, 2 piglets from each pen (n = 8; 4 females and 4 castrated males per treatment) were transported from the Arkell Swine research station to the Animal Science and Nutrition building (University of Guelph), were housed individually and fed phase 2 diets as above; these piglets were given 4 days to adjust to their new environment (Figure 1). On post-weaning d10–12, 8 piglets per dietary treatment were anesthetized and underwent surgery to insert a jugular catheter [8]. Piglets were monitored daily and were allowed 9 days to recover from surgery, during which they had unlimited access to feed and water. On post-weaning d19–21, 8 piglets per day were challenged *i.m.* with 50 µg/kg of LPS from *Escherichia coli* (O55:B5 Sigma-Aldrich, Oakville, Ontario). Blood samples (4 mL) were collected into serum collection tubes (BD vacutainer, Mississauga, ON, Canada) and plasma collection tubes (BD vacutainer containing sodium heparin 185 USP units) before LPS challenge, and 2, 5, 10, 15, and 30 min, and 1, 2, 3, 4, and 5 h post-LPS challenge. Plasma samples were placed on ice and serum samples were left to clot for 1 h before being centrifuged at 1000 × *g* for 25 min. Plasma and serum were aliquoted into 2 mL vials and stored at −80 °C for further analyses. Rectal temperature was recorded before LPS challenge and hourly until 5 h post-LPS challenge to monitor the fever response. Piglets were euthanized using 2.5 mL of pentobarbital administered *i.v.* immediately following the 5-h blood collection.

Total serum cortisol was analyzed 0, 2, 3, and 4 h post-LPS challenge using a commercially available immunoassay (DetectX Cortisol Enzyme Immunoassay, Arbor Assays, MI, USA). Briefly, 50 µL of serum was added to each well in a 96-well plate, as well as 25 µL of cortisol conjugate and 25 µL of cortisol antibody, and the plate was incubated on a shaker for 1 h at room temperature (RT). Each plate was then washed 4 times with 300 µL of wash buffer before adding 100 µL of tetramethylbenzidine substrate to each well and incubating for 30 min at RT. Following this, 50 µL of stop solution was added to each well. Plates were read at 450 nm in a plate reader (Victor^3^ 1420 Multilabel Counter, Perkin Elmer, Wellesley, MA, USA). All samples were run in triplicate and a standard curve was included in each plate. The inter-assay coefficient of variation (CV) was 7.1%.

Plasma chromogranin A (CGA) levels were measured 0, 15, 30, and 60 min post-LPS immune challenge by enzyme-linked immunosorbent assay (ELISA). Briefly, 96-well plates were coated with 5 µg/mL coating antibody and incubated for 24 h at 4 °C. Following antigen coating, plates were washed 3 times with 0.05% Phosphate Buffered Saline-Tween 20 wash buffer. Plates were incubated at RT with 200 µL/well of blocking solution (Invitrogen, Burlington, ON, Canada) for 2 h, washed 3 times with wash buffer and then 100 µL/well of the plasma samples were incubated on a shaker for 2 h at RT. After plasma incubation, plates were washed 3 times with wash buffer, and 100 µL/well of primary detection antibody (sheep anti-human IgG; Novus Biologicals, Oakville, ON, Canada) was added to each well and incubated on a shaker for 2 hat RT, before washing again and incubating with conjugated antibody (rabbit anti-sheep IgG; Novus Biologicals, Oakville, ON, Canada) for 1 h at RT. After a final wash, 100 µL/well of alkaline phosphatase yellow substrate (Sigma-Aldrich, Oakville, ON, Canada) was added to each well and incubated for 20 min at RT in the dark. A standard curve, as well as positive and negative control samples, were added to each plate. Following the 20-min substrate incubation, plates were analyzed at 405 nm in a plate reader (Victor^3^ 1420 Multilabel Counter, Perkin Elmer, Wellesley, MA, USA). The inter-assay CV was 14.5%.

Serum adrenocorticotropic releasing hormone (ACTH) was measured using a commercially available porcine ACTH ELISA kit (Cusabio Biotech LTD, Houston, TX, USA). Analysis was conducted 0, 1, and 2 h post-LPS immune challenge. Briefly, 50 µL of sample was incubated in a 96-well plate with 50 µL of conjugate for 1 h at 37 °C. Following this incubation, the plate was washed 3 times with 200 µL/well of wash buffer before incubation with 50 µL of horseradish peroxidase-avidin solution for 30 min at 37 °C. After washing 3 times with wash buffer as before, 50 µL of substrate A and 50 µL of substrate B were added to each well and incubated in the dark for 15 min at 37 °C. After the 15-min incubation, 50 µL of stop solution was added to each well before reading at 450 nm using a plate reader (Victor^3^ 1420 Multilabel Counter, Perkin Elmer, USA). The inter-assay CV was 4.63%.

A panel of serum cytokines (TNF-α, IL-1β, IL-6, and IL-10) was analyzed using a multiplex assay (Milliplex Map Porcine cytokine/chemokine magnetic bead panel, Millipore, Toronto, ON, Canada). Briefly, 25 µL of each sample was added to a 96-well plate with 25 µL of antibody-immobilized beads. Plates were sealed, wrapped in aluminum foil, and incubated on a plate shaker for 24 h at 4 °C. After 3 washes with wash buffer, detection antibodies were added at a volume of 50 µL per well. The plates were sealed, wrapped in foil, and incubated for 2 h at RT. Following this incubation, plates were decanted, 50 µL of Streptavidin–Phycoerythrin was added to each well, and plates were incubated for 30 min, and then washed thrice. Finally, 100 µL of Luminex^®^ sheath fluid was added, and beads were resuspended on a shaker for 5 min before reading on a Luminex^®^ 200 analyzer (Luminex Corp, Toronto, ON, Canada). Cytokine concentrations were determined by comparing samples to standards of known concentration provided by the manufacturer. The inter-assay CV was 5.5%.

### 2.3. Assessment of Antigen-Specific Immunity

Two piglets per pen (n = 8; 4 females and 4 castrated males per treatment) were antigen sensitized *i.m.* on post-weaning d7 with 0.5 mg/mL ovalbumin (OVA; Sigma-Aldrich, ON, Canada) and 0.5 mg/mL *Candida albicans* cellular antigen (CAA; Greer Laboratories Inc., Lenoir, NC, USA) dissolved in 1 mL of saline containing 0.5 mg/mL of Quil-A adjuvant (Sigma-Aldrich, ON, Canada). On post-weaning d21, piglets received a secondary booster containing the same concentrations of OVA and CAA. On post-weaning d35, piglets were challenged *s.c.* with 0.1 mL of OVA or CAA at a concentration of 1 mg/mL on each of the inner thighs, with a saline control site on each leg. Skin-fold thickness measurements were taken in triplicate to assess the dermal hypersensitivity response (DHR) using Harpenden Skin-fold Calipers (Creative Health Products, Ann Arbor, MI, USA) before and 6, 24, and 48 h post-antigen challenge.

Blood was also collected from the antigen-sensitized piglets on post-weaning d7, d21, and d35 in 10 mL serum collection tubes (BD vacutainer, Mississauga, ON, Canada). Samples were left to clot for 1 h, then centrifuged at 1000 × *g* for 20 min. Serum aliquots were stored at −80 °C until antigen-specific IgG1 and IgG2 analyses were carried out by ELISA. For the ELISAs, plates were coated with 1.4 µg/mL of either OVA, or CAA, and incubated at 4 °C for 48 h. Following antigen coating, plates were washed 5 times with 0.05% PBS-Tween 20 wash buffer. Plates were incubated at RT with 200 µL/well of blocking solution (Bio-Rad Laboratories, Mississauga, ON, Canada) for 1 h, washed 5 times with wash buffer, and then 100 µL/well of serum sample dilutions (1:50 for IgG2 analysis, 1:800 for IgG1 analysis) were incubated in duplicate for 2 h. After washing, 100 µL/well of primary IgG1 or IgG2 antibodies (mouse anti-pig IgG1 and IgG2; Bio-Rad Laboratories, Mississauga, ON, Canada) were added to each well and incubated for 1 h at RT, then washed again and incubated with conjugated antibodies (goat anti-mouse IgG; Sigma-Aldrich, Oakville, ON, Canada) for 1 h. After a final wash, 80 µL/well of alkaline phosphatase yellow substrate (Sigma-Aldrich, Oakville, ON, Canada) was added to each well and incubated for 30 min at RT in the dark. A standard curve, as well as positive and negative control samples, were added to each plate. Following the 30-min substrate incubation, plates were analyzed at 405 nm using a plate reader (Victor^3^ 1420 Multilabel Counter, Perkin Elmer, Wellesley, MA, USA); the inter-assay CV was 1% and 12.7% for IgG1 and IgG2 analyses, respectively.

### 2.4. Fatty Acid Analysis

Plasma samples from 24 piglets (n = 8 per treatment, 4 females and 4 castrated males per treatment), from post-weaning d21 were collected and sent to the University of Guelph Lipid Analytical Lab (Guelph, ON, Canada) for free fatty acid analysis. Total lipids were extracted from 200 µL of sample using the Folch Method [9]. The plasma phospholipid fraction was separated from the neutral lipids by thin-layer chromatography. The fatty acid methyl esters were prepared from the isolated phospholipid fraction and analyzed on a Varian 3400 gas–liquid chromatograph (Palo Alto, CA, USA) with a 60 m DB-23 capillary column (0.32 mm internal diameter, FID detection). Fatty acid standards and mixtures thereof (Nu Chek Prep, Elysian, MN, USA) were used to ensure quantitative and qualitative accuracy and recovery.

### 2.5. Statistical Analysis

Statistical analysis was conducted using PROC GLIMMIX of SAS version 9.4 (SAS Inst. Inc., Cary, NC, USA). Performance data used pen as experimental unit, and pigs that were challenged with LPS were excluded from this data set. The statistical model included pig within pen as a random variable and dietary treatment as a fixed effect. Repeated measures analyses using pig as the experimental unit were used for the LPS immune stress challenge, DHR response, and antigen-specific IgG1 and IgG2 response data. The statistical model used pen as a random effect, treatment (AL, FO, CON) as a fixed effect, and accounted for sex, litter, and sampling time point as well as their interactions. A simplified model was used where fixed effects and their interactions were not significant. A multiple means comparison was used for the fatty acid data, using individual pigs as the experimental unit. The statistical model used litter as a random effect and dietary treatments and sex as fixed effects. Least squared means (LSM) were obtained for all variables, and linear and quadratic contrasts were used to observe trends over time for results from the DHR response and LPS immune stress challenge. Significant differences were reported at *p* < 0.05, and trends were between 0.05 < *p* < 0.1.

## 3. Results

During the study, 10 pigs were removed due to illness unrelated to the trial. For the LPS challenge, 6 pigs (3 AL, 2 FO, and 1 CON) were removed due to difficulties with the jugular catheter, leaving 18 pigs (5 AL, 6 FO, and 7 CON) for the LPS challenge. In addition, 4 pigs (1 AL and 3 CON) were removed from the antigen-specific immunity study due to illness or human error. Two male pigs and 1 female pig were lost from the CON treatment, and one female pig was lost from the AL treatment.

### 3.1. Pigs’ Performance

There were no differences for ADG among any of the treatments over any phase of the trial. Body weights did not differ across treatments throughout the trial. In phase 1, ADFI was increased in pigs fed with FO compared to those fed with CON and AL (*p* < 0.05; Table 3) diets. During feeding phase 2, ADFI was greater for pigs fed the CON versus those fed the FO (*p* < 0.05) and AL (*p* < 0.05) diets. In feeding phase 3, piglets fed the FO diet had reduced ADFI versus those fed the AL and CON diets (*p* < 0.05); however, over the entire post-weaning period, no differences in ADFI were observed. No differences in G:F were observed in any phase of the trial or overall, despite changes in feed intake.

### 3.2. Assessment of Acute-Phase Response to LPS Immune Stress Challenge

**Figure 2:**
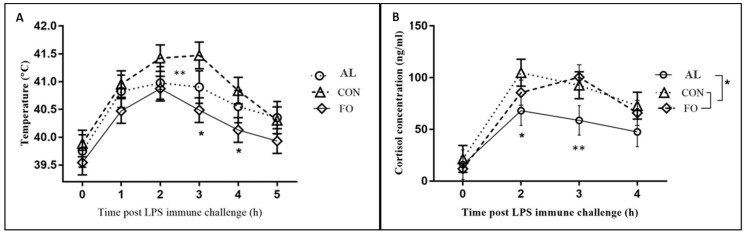
(**A**) Fever response and (**B**) serum cortisol response following piglet LPS immune challenge in pigs fed diets supplemented with microalgae (AL, n = 8), fish oil (FO, n = 8) or a corn oil control diet (CON, n = 6). Results presented as LSM ± SEM. * Significant differences (*p* < 0.05) compared to CON treatment; ** trends (*p* < 0.1).

Pigs fed the CON diet tended to have a greater rectal temperature by 3 h after LPS immune challenge compared to pigs fed the AL diet (*p* < 0.1) and had a significantly greater temperature than pigs fed with the FO at 3 and 4 h after LPS immune challenge (*p* < 0.05, Figure 2A).

**Figure 3:**
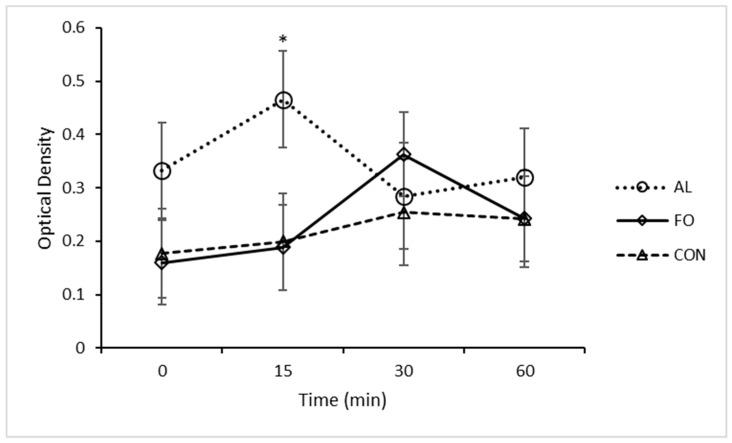
Plasma chromogranin A levels over time following an LPS immune challenge in piglets fed with diets containing microalgae (AL, n = 8), fish oil (FO, n = 8), or fed a corn oil control diet (CON, n = 6). Results are presented as LSM ± SEM. * Significant differences (*p* < 0.05).

Neuroendocrine biomarkers were significantly altered in response to LPS, indicating that the animals responded to the LPS challenge. A significant increase in cortisol concentration was observed at 2 h (*p* < 0.05) post-LPS injection in pigs fed the CON diet compared to pigs fed the AL diet (Figure 2B), and tended to be increased in pigs fed the FO and CON diets compared to those fed the AL diet at 3 h (*p* < 0.1). No differences in serum cortisol concentration were found between pigs fed the FO and CON diets. Plasma CGA concentrations were increased in pigs fed the AL diet versus those fed the FO (*p* < 0.05) and CON (*p* < 0.05) diets 15 min after LPS challenge, but no treatment differences were detected after 30 or 60 min (Figure 3). Serum ACTH concentration tended to be greater for pigs fed the AL diet versus those fed the FO diet before LPS challenge (*p* < 0.1; data not shown), but was not different among dietary treatments after LPS immune stress challenge.

**Figure 4:**
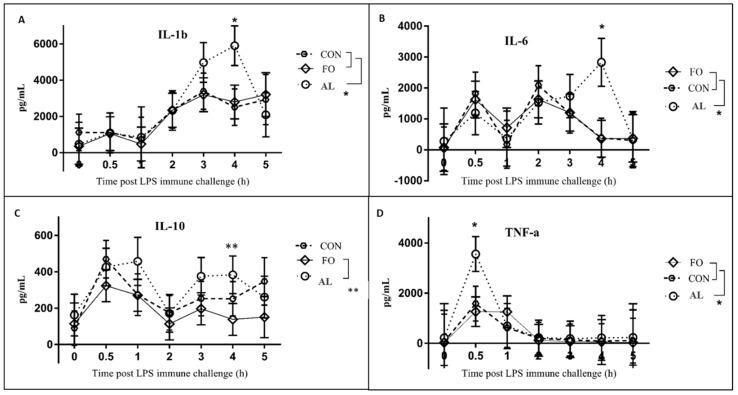
Expression of serum (**A**) IL-1β (**B**) IL-6, (**C**) IL-10 and (**D**) TNF-α, over time in piglets fed with diets supplemented with microalgae (AL, n = 8), fish oil (FO, n = 8), or corn oil control (CON, n = 6). Results are presented as LSM ± SEM. *Significant differences (*p* < 0.05) between treatments at a time point are denoted with a single asterisk. ** Trends (*p* < 0.1) are denoted with a double asterisk.

Cytokine biomarkers were also affected by dietary treatment; serum concentrations of IL-1β (*p* < 0.05; Figure 4A), IL-6 (*p* < 0.05, Figure 4B) were significantly greater, and concentrations of IL-10 tended to be greater (*p* < 0.1, Figure 4C) 4 h after LPS immune challenge for pigs fed the AL diet compared to those fed the FO and CON diets. Concentrations of TNF-α (Figure 4D) were significantly greater in pigs fed the AL versus those fed the FO and CON (*p* < 0.05) diets 0.5 h post-LPS challenge.

### 3.3. Assessment of Antigen-Specific Immunity

**Figure 5:**
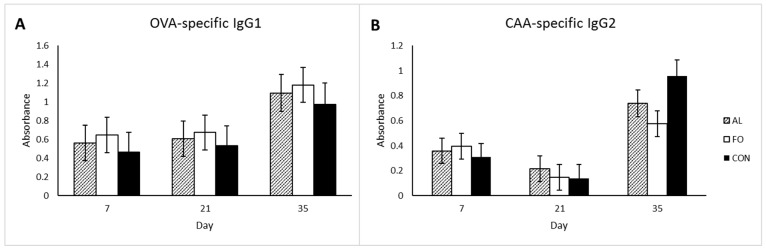
(**A**) OVA-specific IgG1 serum antibody levels and (**B**) CAA-specific serum IgG2 antibody levels on trial days 7, 21, and 35 in pigs fed diets supplemented with microalgae (AL, n = 8), fish oil (FO, n = 8), or corn oil control (CON, n = 8). Results are presented as LSM ± SEM.

**Figure 6:**
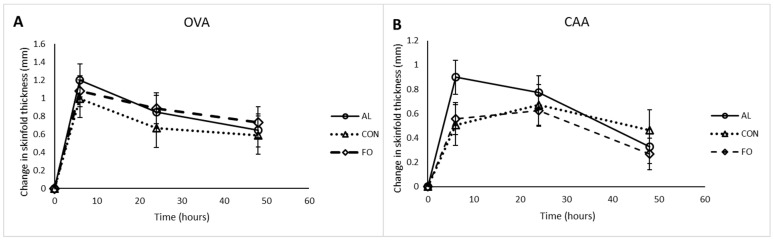
Change in skin-fold thickness in response to (**A**) OVA antigen and (**B**) CAA antigen at 6, 24, and 48 h post-injection in pigs fed with diets supplemented with microalgae (AL, n = 8), fish oil (FO, n = 8), or corn oil control (CON, n = 8). Results are presented as LSM ± SEM.

Assessment of antigen-specific immunity showed that all animals responded to the OVA and CAA antigen immunizations. The OVA-specific IgG1 and CAA-specific IgG2 responses, however, were not different among treatments at any time point (Figure 5). Repeated measures analysis showed an increase in the IgG1 response between d7 and d35 (*p* < 0.05) following antigen sensitization, and a slight decrease in IgG2 concentrations between d7 and d21 post-antigen sensitization (*p* < 0.05), which then increased significantly higher than d7 between d 21 and d35 post-antigen sensitization (*p* < 0.05). No significant differences in the DHR to OVA and CAA antigens were observed among any of the treatment groups (Figure 6) despite a significant increase in skin swelling from baseline levels at 6 and 24 h post-injection.

### 3.4. Fatty Acid Analysis

Results from plasma fatty acid analysis indicated that levels of DHA and EPA were significantly different among treatments (*p* < 0.05, Table 4). Total n-3 PUFA content and the ratio (n-3:n-6) were not different between the FO and AL treatments; however, both were significantly greater in the FO and AL pigs than the CON pigs (*p* < 0.05).

## 4. Discussion

Previously, macroalgae has been used in swine feed to assess the effects on piglets’ growth and gut health, although these algae supplements were used as a source of glucans and polysaccharides and had comparatively low crude fat content [10,11]. Previous studies have also examined the use of AL in livestock feed to promote animal growth and to develop value-added products such as n-3 PUFA-enriched meat and milk [12,13], but the present study is one of the first to investigate AL as a source of n-3 PUFA to promote weanling piglets’ health. FO supplementation has previously been investigated in pigs, and has been shown to attenuate the dermal hypersensitivity response [3], and fever and cytokine responses following *i.p.* and *i.v.* LPS challenge [14,15,16]. The major biomolecules in FO include the n-3, PUFA, DHA, and EPA. Microalgae can also synthesize DHA and/or EPA, depending on the species [12]. Therefore, similar to FO, AL also has immune modulating properties [17,18] and its inclusion in animal diets, particularly during critical periods such as weaning, could help reduce the negative effects of stress on animal health. This may in turn reduce the need for veterinary interventions such as antibiotic treatments and reduce economic losses to producers.

The results obtained from pigs on the FO diet in the present study are in agreement with those from other porcine studies in that FO supplementation attenuated the fever response to LPS immune challenge as compared to the CON diet [14,15]. Fever is typically induced by the production of pro-inflammatory cytokines, such as TNF-α and IL-1β, during LPS immune challenge [19,20]. Despite the attenuated fever response in the FO pigs, concentrations of TNF-α and IL-1β, as well as IL-6 and IL-10 surprisingly did not differ between FO and CON animals up to 5 h post-LPS challenge. However, similar results were observed by Upadhaya et al. [21] where, despite linseed oil supplementation that is rich in the n-3 PUFA α-linolenic acid, no changes in IL-1β and IL-6 were observed between treatments following LPS challenge administered *i.m.* In addition, Luo et al. [22] observed significant decreases in the expression of IL-1β and IL-6 in the longissimus dorsi muscle of piglets fed diets containing 7% FO, whereas these cytokines significantly increased in spleens of piglets from the same study. Therefore, while it is typically thought that FO exerts anti-inflammatory effects due to n-3 PUFA concentrations, cytokine concentrations in response to FO supplementation appear variable in different tissues.

In pigs fed the AL-supplemented diet, an attenuated fever response was also seen in comparison to CON pigs, although it was not considered statistically significant. The AL pigs also had significantly reduced cortisol levels in response to LPS immune challenge in comparison to both FO and CON pigs. The attenuated AL cortisol response is supported by a recent rat study [17], in which dietary supplementation with algae oil was used throughout pregnancy, lactation, and after weaning. The authors found a significantly reduced corticosterone response to acute stress (forced swimming test) in male offspring compared to male offspring from the control treatment [17].

The AL-supplemented pigs also had a unique LPS-induced cytokine profile in comparison to the other treatment groups. The production of TNF-α at 30 min, and IL-1β and IL-6 at 4 h post-LPS immune challenge was significantly higher in the AL treatment as compared to FO and CON treatments. Unexpectedly, these increased cytokine levels are not consistent with the attenuated fever and cortisol responses in the AL pigs at corresponding time points, particularly as these cytokines are known to induce fever [19,20], and the production of cortisol during immune challenge [23]. A study conducted by Paschoal et al. [24] found that treatment of rat neutrophils with DHA resulted in increased cytokine production in response to LPS stimulation compared to neutrophils treated with EPA. In the present study, it is possible that the elevated cytokine levels in the AL treatment are due to the low amounts of EPA and relatively high amounts of DHA in the AL diet. This suggests that while AL appears to attenuate the overall cortisol response, it increases cytokine production that drives the fever response.

It is possible that the observed results may be influenced by both the LPS dose and the route of administration. A previous study comparing LPS administered *i.v.*, *i.m*., *s.c.*, and *i.p.* noted that the route of administration drastically affected both the kinetics and the amplitude of the fever response in rabbits [25]. It has also been observed that concentrations of LPS up to 100-fold greater may be required to elicit similar responses among different routes of administration [25]. It is therefore also possible that the route of administration and dose both affect the subsequent cortisol and cytokine responses following LPS challenge. However, due to the lack of data pertaining to AL supplementation in conjunction with LPS immune challenge, it is unknown at this time if the results obtained, particularly in AL-supplemented pigs, would differ with alternate routes of LPS administration, and therefore warrants further investigation. Presently, LPS was administered *i.m.* to help prepare a non-invasive protocol for future LPS challenges, where *i.v.* administration would not be feasible.

The findings of the present study following the LPS immune challenge were much different than originally anticipated, and may possibly be attributed to the quick-acting sympatho-adrenomedullary (SAM) response, and the so-called inflammatory reflex [26,27]. It was interesting to observe that although basal levels of ACTH were higher in the AL treatment, ACTH was not induced by the *i.m.* LPS immune challenge; thus, it is possible that downstream cortisol production was stimulated by LPS-induced splanchnic nerve activity to the adrenal cortex rather than pituitary ACTH [28]. Likewise, direct sympathetic innervation of the adrenal medulla and subsequent release of catecholamines may also limit the cytokine, fever, and cortisol responses during LPS immune challenge. To assess this, plasma CGA, as an indirect marker of SAM activity was measured. Chromogranin-A is a protein found within endocrine and neuroendocrine secretory vesicles, and its concentration has been found to correlate with catecholamine release [29]; as such, it has been considered an alternative indirect stress biomarker in pigs [30]. CGA concentrations have previously been measured in saliva [30,31], and therefore requires validation as a potential stress biomarker in other tissues including plasma. Interestingly, the AL-supplemented pigs had a unique CGA response during LPS immune challenge, with significantly higher plasma levels 15 min post-LPS challenge in comparison to the FO and CON pigs. Previous studies examining salivary CGA levels in swine demonstrated a rapid increase in CGA in response to stressors including co-mingling, feed deprivation, and restraint [30,31]. While treatment differences were observed, plasma CGA concentrations did not significantly change over time in the CON treatment during LPS immune challenge. This suggests that plasma CGA may not be a suitable biomarker of LPS immune stress. It is possible that the route of LPS administration could potentially also differentially affect plasma CGA levels, and this should be further explored.

Stressful events affect not only the hypothalamic–pituitary–adrenal (“stress”) axis, but also the immune system, which can directly affect the health of animals. For example, elevated cortisol levels can alter the ratio of CD4^+^/CD8^+^ T-lymphocytes, which can increase animal susceptibility to disease [32]. As the experimental diets in the present study were meant to support healthy immune function, the DHR and IgG1 and IgG2 levels were monitored following booster vaccination with two different types of antigens to assess the status of the acquired immune system. No significant changes among dietary treatments were observed for the measured immune response parameters. CAA-specific IgG2 levels decreased from d7 to d21 but then increased by d35, which was contrary to our hypothesis. This could be due to production of IgM in response to primary vaccination, with immunoglobulin class-switching to IgG2 following the secondary vaccination. However, overall there were no treatment effects on acquired immune response.

Although the AL and FO experimental diets from this study were matched for the total n-3 PUFA content, the content of long-chain fatty acids, EPA and DHA could have influenced the findings of this study. While AL and FO are both rich in anti-inflammatory and immunomodulatory n-3 PUFA, DHA is the major n-3 PUFA in AL, whereas, FO has significant amounts of EPA and a greater ratio of EPA:DHA than AL; EPA has been shown to attenuate the fever response in rabbits following immune challenge with poly(I:C) [33]. Also, since the AL supplement that was used in the present study was a whole algae product, the observed treatment differences may have been influenced by other bioactive compounds found in the AL. This includes molecules such as fat-soluble vitamins, carotenoids, and β-glucans, which are known to have immune-altering properties [13,34]. Finally, certain algae species have also been found to have anti-nutritional factors such as lectins, tannins, phytic acid, and protease inhibitors [7], that could have contributed to the treatment differences observed in this study, although no differences in growth were observed.

A previous study has shown that there were no negative effects on final market weight and performance parameters of pigs fed with low-quality protein diets [5]. However, diets with low-quality protein sources have been found to have a negative impact on pigs’ performance when sub-clinical infections are present [5]. Inclusion of FO in diets with low-quality protein sources can overcome the negative effects on performance caused by sub-clinical infection [15]. In the present study, while AL or FO supplementation did not affect ADG of pigs, despite the enriched levels of n-3 PUFA in plasma samples, reduced feed intake in FO and AL pigs in phase 2 and reduced feed intake in FO pigs in phase 3 may help to decrease overall feeding costs. Further studies should be conducted following piglets fed with FO or AL in the nursery phase to final market weight to determine if the changes in feed intake in phase 2 and phase 3 are consistent throughout the entire production cycle, and to determine if any other changes in immune status or stress response are observed leading up to slaughter.

## 5. Conclusions

AL and FO are both important dietary sources of n-3 PUFA, which may help promote normal growth and health in weanling piglets. Both AL and FO appeared to reduce fever response to LPS immune challenge in contrast to pigs fed with CON. While AL reduced cortisol levels in response to LPS, cytokine levels (IL-1β, IL-6, IL-10, and TNF-α) were elevated compared to FO and CON animals. Neither dietary treatment had any effect on acquired immune function, contrary to what was hypothesized. Although inclusion of AL and FO in nursery diets with low-quality protein sources influenced feed intake in the differing feeding phases, there were no overall effects of AL and FO on either feed intake or feed efficiency. Taken together, these results provide support for AL as an alternative dietary supplement to fish-oil supplements.

## Figures and Tables

**Table 1:** Crude fat, DHA, EPA, and total omega-3 content of the microalgae and fish oil supplements used in the experimental diets.

Ingredient Composition (%)	Microalgae	Fish Oil
Crude fat	70.00	87.03
DHA ^1^	27.72	16.62
EPA ^2^	0.36	17.40
Total omega-3	28.69	42.45

^1^ DHA, docosahexanoic acid; ^2^ EPA, eicosapentanoic acid.

**Table 2:** Formulations of experimental diets.

	Phase 1			Phase 2			Phase 3
Days on Feed	0–7			7–21			21–42
Ingredient (%)	Control	1.25% FO ^1^	3.12% AL ^2^	Control	1.25% FO ^1^	3.12% AL ^2^	Common
Soybean meal	30.00	30.00	30.00	35.00	35.00	35.00	30.00
Corn (NRC ^3^; 8.3%CP ^4^)	45.28	45.28	43.42	41.00	41.00	39.14	48.82
Wheat	15.00	15.00	15.00	15.00	15.00	15.00	15.00
Monocalcium phosphate	1.52	1.52	1.52	1.24	1.24	1.24	0.97
Limestone	1.30	1.30	1.30	1.25	1.25	1.25	1.17
Salt	0.50	0.50	0.50	0.40	0.40	0.40	0.40
Mineral/Vitamin premix ^5^	0.60	0.60	0.60	0.60	0.60	0.60	0.60
L-Lysine	0.37	0.37	0.37	0.26	0.26	0.26	0.35
L-Methionine	0.15	0.15	0.15	0.11	0.11	0.11	0.09
L-Threonine	0.24	0.24	0.24	0.12	0.12	0.12	0.10
L-Tryptophan	0.02	0.02	0.02	–	–	–	–
Vitamin E	0.02	0.02	0.02	0.02	0.02	0.02	–
Microalgae ^6^	–	–	3.12	–	–	3.12	–
Fish oil	–	1.25	–	–	1.25	–	–
Corn oil	5.00	3.75	3.75	5.00	3.75	3.75	2.50
Calculated nutrient composition ^7^							
Metabolizable energy, kcal/Kg	3472	3471	3472	3479	3478	3479	3358
Net energy, kcal/Kg	2612	2611	2636	2592	2591	2602	2509
CP (%)	20.85	20.85	20.69	22.65	22.65	22.5	20.97
Crude fat (ether extract, %)	7.06	6.96	7.42	6.99	6.89	7.35	4.81
SID ^8^ Lysine (%)	1.21	1.21	1.21	1.25	1.25	1.25	1.2
SID Methionine + cysteine (%)	0.71	0.71	0.71	0.72	0.72	0.71	0.66
SID Threonine (%)	0.86	0.86	0.86	0.81	0.81	0.81	0.73
SID Tryptophan (%)	0.24	0.24	0.24	0.25	0.25	0.25	0.23
Analzed nutrient composition (%)							
Dry matter	92.27	92.04	92.11	91.87	92.55	92.23	na
CP	17.70	18.53	20.31	22.02	21.62	22.61	na
Crude fat	5.22	5.69	7.02	6.11	6.96	7.05	na
Calcium	0.82	0.96	0.88	0.81	0.86	0.88	na
Phosphorus	0.74	0.76	0.73	0.69	0.74	0.73	na
Sodium	0.18	0.20	0.19	0.15	0.18	0.16	na
Potassium	0.86	0.88	0.92	1.02	0.98	1.05	na
Magnesium	0.17	0.18	0.18	0.19	0.19	0.20	na

^1^ FO, Fish oil; ^2^ AL, algae; ^3^ NRC, National Research Council 2012; ^4^ CP, crude protein; ^5^ supplied per kg of diet: vitamin A, 12,000 IU as retinyl acetate; vitamin D3, 1,200 IU as cholecalciferol; vitamin E, 48 IU as DL-α-tocopherol acetate; vitamin K, 3 mg as menadione; vitamin B12, 0.03 mg; pantothenic acid, 18 mg; riboflavin, 6 mg; choline, 600 mg; folic acid, 2.4 mg; niacin, 30 mg; thiamine, 18 mg; pyridoxine, 1.8 mg; biotin, 200 µg; Cu, 18 mg as CuSO_4_·5H_2_O; Fe, 120 mg as FeSO_4_; Mn, 24 mg as MnSO_4_; Zn, 126 mg as ZnO; Se, 0.36 mg as FeSeO_3_; I, 0.6 mg as KI; DSM, Ayr, ON, Canada; ^6^ microalgae supplied by Alltech Inc., and supplied as a dried biomass, containing 15.8% CP, 70% crude fat and 17% DHA; ^7^ calculated on the basis of the NRC (2012) ingredient values; ^8^ SID, standardized ileal digestible.

**Table 3:** Body weights, average daily gain (ADG), average daily feed intake (ADFI) and gain-to-feed ratio for pigs from weaning to end of phase 3 fed diets supplemented with either 3.12% microalgae (AL; n = 48) or 1.25% fish oil (FO; n = 48), or 5% corn oil (CON; n = 48). Results presented as least squared means (LSM) ± standard error of the means (SEM).

	CON	FO	AL	SEM	P-Value ^1^
**Body Weight**, kg					
Initial	6.86	6.91	6.82	0.25	0.97
Day 7	7.23	7.41	7.13	0.29	0.79
Day 21	12.05	12.01	11.82	0.44	0.92
Day 35	21.88	22.09	22.30	0.77	0.92
**ADG**, g					
Phase 1	61	83	60	13	0.35
Phase 2	382	369	358	18	0.65
Phase 3	630	630	655	40	0.88
Overall	442	436	441	19	0.97
**ADFI**, g					
Phase 1	180 ^b^	207 ^a^	172 ^b^	6	<0.0001
Phase 2	492 ^a^	463 ^b^	445 ^b^	9	0.0005
Phase 3	953 ^a^	848 ^b^	962 ^a^	37	0.02
Overall	623	608	600	11	0.32
**G:F**					
Phase 1	0.30	0.40	0.34	0.063	0.52
Phase 2	0.76	0.79	0.81	0.044	0.75
Phase 3	0.63	0.79	0.68	0.078	0.37
Overall	0.69	0.76	0.72	0.042	0.44

^1^*p*-value for the main effect of dietary treatment; ^a,b^ Significant differences (*p* < 0.05) are indicated by differing superscripts within a row.

**Table 4:** EPA, DHA, total n-3, and ratio of n-3:n-6 in phase 1 and phase 2 diets and in plasma from pigs fed with diets supplemented with 3.12% microalgae (AL; n = 8), 1.25% fish oil (FO; n = 8), or 5% corn oil (CON; n = 8) diets. Data from dietary fatty acid analysis presented as raw data; results from plasma fatty acid analysis presented as LSM ± SEM.

Treatment	AL	FO	CON
**Phase 1 diets**, % total fatty acids			
EPA ^1^	0.36	1.66	0.05
DHA ^2^	5.32	1.52	0.00
Total n-3 ^3^	7.53	5.92	2.00
Ratio n3:n6 ^4^	0.16	0.12	0.04
**Phase 2 diets**, % total fatty acids			
EPA	0.26	2.00	0.07
DHA	5.71	1.87	0.33
Total n-3	8.01	6.89	2.36
Ratio n3:n6	0.18	0.14	0.04
**Piglet plasma**, % total fatty acids			
EPA	1.74 ± 0.34 ^b^	6.00 ± 0.34 ^a^	0.16 ± 0.34 ^c^
DHA	5.76 ± 0.26 ^a^	2.93 ± 0.26 ^b^	0.00 ± 0.26 ^c^
Total n-3	8.95 ± 0.57 ^a^	11.23 ± 1.12 ^a^	0.12 ± 1.73 ^b^
Ratio n-3:n-6	0.21 ± 0.017 ^a^	0.29 ± 0.033 ^a^	0.018 ± 0.051 ^b^

^1^ EPA, eicosapentanoic acid; ^2^ DHA, docosahexanoic acid; ^3^ total n-3, total omega-3 polyunsaturated fatty acids; ^4^ ratio n-3:n-6, ratio of total omega-3 polyunsaturated fatty acids to total omega-6 polyunsaturated fatty acids; ^a,b,c^ differing letters across rows indicate significant differences (*p* < 0.05) between treatments.
